# Thyroid cancer risk in Belarus among children and adolescents exposed to radioiodine after the Chornobyl accident

**DOI:** 10.1038/sj.bjc.6605967

**Published:** 2010-11-23

**Authors:** L B Zablotska, E Ron, A V Rozhko, M Hatch, O N Polyanskaya, A V Brenner, J Lubin, G N Romanov, R J McConnell, P O'Kane, V V Evseenko, V V Drozdovitch, N Luckyanov, V F Minenko, A Bouville, V B Masyakin

**Affiliations:** 1 Department of Epidemiology and Biostatistics, University of California San Francisco, 3333 California Street, Suite 280, San Francisco, CA 94118, USA; 2 Division of Cancer Epidemiology and Genetics, NCI/NIH/DHHS, Bethesda, MD 20892, USA; 3 The Republican Research Center for Radiation Medicine and Human Ecology, Gomel 246040, Belarus; 4 The Thyroid Center, Columbia University, New York, NY 10032, USA; 5 Radiology Department, Thomas Jefferson University Hospital, Philadelphia, PA 19107, USA; 6 Belarusian Medical Academy of Post-Graduate Education, Minsk 220714, Belarus

**Keywords:** thyroid neoplasms, iodine radioisotopes, Chernobyl nuclear accident, risk, iodine deficiency

## Abstract

**Background:**

Previous studies showed an increased risk of thyroid cancer among children and adolescents exposed to radioactive iodines released after the Chornobyl (Chernobyl) accident, but the effects of screening, iodine deficiency, age at exposure and other factors on the dose–response are poorly understood.

**Methods:**

We screened 11 970 individuals in Belarus aged 18 years or younger at the time of the accident who had estimated ^131^I thyroid doses based on individual thyroid activity measurements and dosimetric data from questionnaires. The excess odds ratio per gray (EOR/Gy) was modelled using linear and linear–exponential functions.

**Results:**

For thyroid doses <5 Gy, the dose–response was linear (*n*=85; EOR/Gy=2.15, 95% confidence interval: 0.81–5.47), but at higher doses the excess risk fell. The EOR/Gy was significantly increased among those with prior or screening-detected diffuse goiter, and larger for men than women, and for persons exposed before age 5 than those exposed between 5 and 18 years, although not statistically significant. A somewhat higher EOR/Gy was estimated for validated pre-screening cases.

**Conclusion:**

10–15 years after the Chornobyl accident, thyroid cancer risk was significantly increased among individuals exposed to fallout as children or adolescents, but the risk appeared to be lower than in other Chornobyl studies and studies of childhood external irradiation.

Convincing evidence exists of an association between exposure to external radiation and an increased risk of thyroid cancer (Ron and Schneider, 2006), but risks from internally deposited radioactive iodines were not well studied until recently. The Chornobyl (Chernobyl) nuclear power plant accident resulted in exposure of populations in Ukraine, Belarus and the Russian Federation to large amounts of radionuclides (chiefly ^131^I, ^133^I and ^137^Cs) (UNSCEAR, 2000). Those exposed as children or adolescents received comparatively high radiation doses due to the small size of thyroid gland and high consumption of milk contaminated with radioactive iodines (Gavrilin *et al*, 2004).

An increase in thyroid cancer among exposed children and adolescents in the contaminated areas has been reported since the accident (Kazakov *et al*, 1992; Astakhova *et al*, 1998; Jacob *et al*, 1999). Questions, however, have been raised about the potential effects of screening and case ascertainment as well as modifying effects of iodine deficiency on risk estimates (Boice, 2005). The modifying effects of gender, age at exposure, and time since exposure (Ron, 2007) and their differences and similarities with the risks observed after external irradiation (Shore, 1992; Ron and Schneider, 2006) are not well understood.

We initiated two screening studies of children and adolescents exposed to Chornobyl fallout in Ukraine and Belarus using similar designs to clarify these unresolved issues (Stezhko *et al*, 2004). In the Ukrainian study, we estimated an excess odds ratio per gray (EOR/Gy) of 5.25 (95% confidence intervals (CIs): 1.70–27.5) for prevalent thyroid cancer detected during initial screening (Tronko *et al*, 2006). In this report, we evaluated the dose–response for prevalent thyroid cancers diagnosed during the first round of screening in Belarus. We also present an analysis of validated self-reported pre-screening cases.

## Materials and methods

A detailed description of the study population and methods has been published previously (Stezhko *et al*, 2004). We identified 38 543 individuals aged 18 years or younger at the time of the Chornobyl accident on 26 April 1986 who had thyroid radioactivity measurements taken in Belarus within 2 months after the accident. We attempted to trace these individuals through local and regional address bureaus, military registration offices, departments of education and public health, and medical establishments. Approximately 5% (*n*=1804) of the potential study subjects were ineligible (incorrect age, died, incarcerated, moved out of the country, etc.) and 20 526 (53.3%) could not be traced. Of the 16 213 individuals who were traced and invited to participate in the study, 11 970 (73.8%) were screened for thyroid diseases (11 903 in 1996–2001 and 67 in 2002–2004). During screening, 202 individuals were determined to be ineligible due to incorrect age (*n*=114), incorrect identification (*n*=20), or lack of thyroid tissue due to primary thyroid gland aplasia (*n*=10) or surgery for benign thyroid condition (*n*=58). An additional 104 subjects were excluded because their doses could not be estimated adequately. For the main analyses, we also excluded the 53 subjects who had a prior thyroid cancer, leaving a total of 11 611 subjects.

The study was approved by institutional review boards in Belarus and the United States. Informed consent was provided by the study participants or by accompanying guardians for minors.

The majority of study subjects resided in Minsk and Gomel oblasts (an oblast is an administrative subdivision similar in size to a state or province) and were screened at study centres in Minsk and Gomel cities or at local medical clinics by visiting mobile screening teams. The initial protocol called for biennial screening, but by decree of the Ministry of Health of Belarus, all persons under the age of 18 years at the time of screening (almost a third of the cohort) were recalled annually for screening examinations. Thyroid screening consisted of ultrasound examination and palpation by a sonographer and a clinical examination with independent palpation by an endocrinologist. Any discrepancies were resolved by a third examination conducted jointly by both doctors. At the time of screening, participants were administered questionnaires to ascertain demographic, residential, dietary and medical history, and blood and spot urine samples were collected.

Patients with thyroid nodules detected either on palpation or ultrasonogram that measured at least 10 mm and all nodules that measured 5–10 mm and were sonographically suspicious for malignancy (hypoechogenic, indistinct border, calcified inclusions, extension through the thyroid capsule or suspicious lymphadenopathy) or with diffusely abnormal thyroid tissue accompanied by unexplained cervical lymphadenopathy were referred to the Minsk and Gomel study centres for further evaluation and ultrasound-guided fine needle aspiration (FNA) biopsy.

Thyroid cancers were included in the analysis if they were surgically removed within 3 years of initial screening. Two study pathologists diagnosed thyroid cancers based on examination of tumour tissue. All diagnoses were confirmed by the Chornobyl Tissue Bank (CTB) or an *ad hoc* international panel of thyroid pathologists. Pre-screening thyroid cancers were defined as cases diagnosed after the accident and reported by subjects at initial screening. Cancers were validated by review of patients’ medical and pathology records.

^131^I represents about 95% of the thyroid dose (Minenko *et al*, 2006), and thyroid doses mainly came from intake of ^131^I-contaminated milk. Thyroid doses, estimated using methodology similar to that used in the Ukrainian study (Likhtarev *et al*, 2006), were based on: (1) direct thyroid measurements; (2) a radioecological model, which was used to assess the temporal variation of ^131^I in the thyroid and (3) personal interviews, which provided information on individual dietary and lifestyle habits that were used to adjust the radioecological model. The results of direct thyroid measurements in Belarus were corrected for contribution to the measured signal from external surface contamination of body and clothes as well as internal contamination of cohort member's body with caesium isotopes. Additional data sources were used to update the parameters of the dosimetry model, including thyroid volume measurements from the Sasakawa Memorial Foundation to derive age-specific thyroid masses (Skryabin *et al*, 2010); measurements of ^131^I in soil to verify the validity of calculated ^131^I deposition density in each settlement; and ^131^I measurements in soil and grass samples to derive an interception factor of ^131^I by vegetation.

### Statistical methods

We analysed the prevalence of thyroid cancer using models for the binomial odds (Breslow and Day, 1987). The dose categories were selected to evenly distribute cases. We fitted an EOR model to continuous doses to estimate the EOR/Gy where the disease odds is: 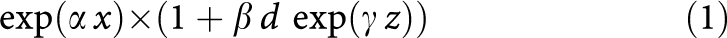 where *d* represents radiation dose, *x* and *z* are covariate vectors representing confounding and effect modification variables, respectively, and *α*, *β* and *γ* are unknown parameters.

Past iodine deficiency was evaluated using proxy indicators, including place of residence at the time of the accident, self-reported history of diffuse goiter, diffuse goiter diagnosed during screening and enlarged thyroid (thyroid volume>18 ml for women and >25 ml for men) (Rasmussen *et al*, 2002). In addition, we evaluated gender, age at the time of the accident, and oblast of residence, urban/rural status and age at screening, history of nodular goiter, and urinary iodine concentration as risk factors and/or possible modifiers of the dose–response. We retained potential confounding variables in the model if they significantly improved the model fit or if the estimate of the EOR changed by more than 10%.

We analysed data using the EPICURE software package (Preston *et al*, 1993). All statistical tests were two-sided with a specified type I error of 0.05, and 95% CIs were estimated by maximum likelihood procedures. Linear trend test was based on the means of the dose categories.

## Results

### Analysis of prevalent cases detected during screening

Of the 595 subjects referred for FNA at the first screening cycle, the referral was cancelled for 18 subjects (3.0%) who did not meet the FNA criteria and FNA was performed for 553 subjects for a compliance rate of 95.8%. Of the 167 subjects referred for surgery, 152 complied (91.0%). Additional three surgeries were performed outside the study, but their pathology slides were reviewed by the study pathologists. All but one of the 87 thyroid cancers were papillary carcinomas (1 was follicular carcinoma). Nine cases had two or more separate thyroid cancers in different locations and thirteen cases had simultaneous diagnoses of thyroid cancer and follicular adenoma. Of the 87 cases, 5 were discovered during surgery for benign thyroid conditions.

Adjusting for radiation dose, background thyroid cancer ORs were non-significantly higher for women compared to men (OR=1.40, *P*=0.12) and older ages at screening (*P*=0.19; Table 1). The prevalence rate was significantly higher for individuals with a self-reported history of nodular (OR=23.21, *P*<0.001) or diffuse goiter (OR=5.15, *P*<0.001), or with nodular and diffuse goiter diagnosed at screening (ORs=19.79 and 3.16, *P*<0.001). Subjects with a family history of nodular goiter had an increased OR (OR=3.54, *P*<0.001). Oblast of residence at the time of screening, urban/rural status and current urinary iodine concentration levels were not associated with thyroid cancer after adjustment for radiation dose. Gender, age at screening and oblast of residence at the time of screening were independent risk factors of the background thyroid cancer risks and were retained in all further models.

**Figure 1:**
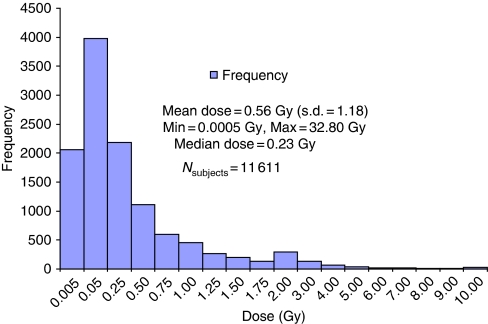
Distribution of thyroid doses in the cohort

**Figure 2:**
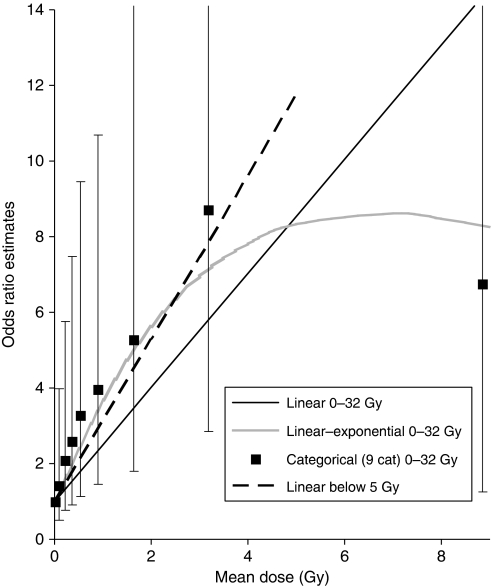
Categorical odds ratios and fitted dose–response lines

Thyroid doses among the 11 611 subjects ranged from nearly 0 to 32.80 Gy (Figure 1) with an arithmetic mean of 0.56 (s.d.=1.18), and differed significantly between cases and non-cases (1.08 *vs* 0.56 Gy, *P*<0.001). Increasing thyroid doses were significantly associated with increased risk of thyroid cancer (*P*_linear trend_<0.001, not shown). When the highest category was split at 5 Gy to reflect a changing dose–response pattern, the linear trend remained highly significant (*P*_linear trend_<0.01; Table 2). A linear model did not fit the data over the full range of doses and the test of no departure from linearity was rejected (*P*=0.05). A linear–exponential model provided an improved fit to the data (Figure 2; estimates *β*=3.11 and *γ*=−0.15 for an OR at 1 Gy of 3.68).

For doses <5 Gy (omitting 118 non-cases, 2 cases), linearity was not rejected (*P*=0.90) and the estimated EOR/Gy was 2.15 (95% CI: 0.81–5.47, *P*<0.001). Further restriction to subjects with doses <1 Gy (omitting an additional 23 cases, 1534 non-cases) resulted in a linear dose–response with an EOR/Gy of 4.92 (95% CI: 1.32–17.12). We limited remaining analyses to subjects with doses <5 Gy. We observed non-significantly higher dose–response estimates for men (*P*=0.08; Table 3). Age at exposure did not significantly modify the radiation dose and thyroid cancer association (*P*=0.48), although risk estimates decreased with increasing age. There was a suggestion of increased dose effects for subjects residing in Gomel oblast at the time of the accident (*P*=0.07). The EOR/Gy was larger in subjects with history of diffuse goiter or diagnosed with diffuse goiter or enlarged thyroid volume at screening compared with those without these conditions (*P*<0.01). Dose effects were unrelated to current urinary iodine concentration (*P*=0.30). Tumours ranged in size from 1 to 47 mm (mean=11 mm, median=9 mm). The estimated dose effects did not differ by tumour size (EOR/Gy=1.35 and 3.40 for 5–10 and ⩾10 mm, respectively, *P*=0.30; Table 3).

### Analysis of pre-screening cases

Self-reported diagnosis of pre-screening thyroid cancer was confirmed for 53 subjects. The majority of the pre-screening thyroid cancers were diagnosed among men (54.7 *vs* 42.5%), and among persons exposed at a younger age (mean age 5.5 years *vs* 8.7) and with a higher mean dose (mean dose 1.77 *vs* 1.08 Gy). They were more likely to live in rural areas, both at the time of the accident and at the time of screening (not shown). Both pre-screening and screening-detected cases had a similar family history of nodular goiter (17.0 and 23.0%), but different self-reported history of diffuse goiter (0 and 10.3%). Similar to the analysis of screening-detected cases, the linear–exponential model provided the best fit to the data on pre-screening cases (estimates *β*=12.87 and *γ*=−0.12 for an OR at 1 Gy of 12.41; not shown). Exclusion of 118 non-cases and 5 pre-screening thyroid cancer cases with doses 5 Gy and above resulted in the linear EOR/Gy of 8.18 (95% CI: 2.11–72.71), *P*<0.001 and the exponential term was no longer significant (*P*=0.43; Table 4). There was no significant effect modification by gender (*P*=0.88), age at exposure (*P*=0.32) or place of residence at the time of the accident (*P*=0.15).

### Combined analysis of pre-screening and screening cases

When we combined pre-screening cases with cases diagnosed during regular screening, the EOR/Gy estimate from the linear model based on doses <5 Gy was 3.16 (95% CI: 1.49–6.95, *P*<0.001; Table 4). The linear slope did not differ significantly between the pre-screening and screening cases (*P*=0.58; not shown). Although there was no significant difference in radiation effects according to gender and age at exposure in the combined cases, the EOR/Gy for those living in Gomel oblast in 1986 was four times the value for those from other oblasts (EOR/Gy=3.93 and 1.00 respectively, *P*=0.04).

## Discussion

Our results from the first screening in the cohort of Belarusian children and adolescents exposed to radioactive iodine fallout from the Chornobyl accident showed a significant dose–response relationship between ^131^I thyroid dose and thyroid cancer, which was linear–exponential across the full range of doses, but consistent with linearity below 5 Gy (EOR/Gy=2.15, 95% CI: 0.81–5.47). There was some indication that men and those exposed at younger ages had larger radiation-associated risks, as measured by EOR/Gy, but they were not statistically significant. Proxy indicators of iodine deficiency enhanced radiation-associated risks of thyroid cancer. Similar effects with higher-point EOR/Gy estimates were observed for pre-screening thyroid cancer cases confirmed through medical records.

A major strength of the study was the individual estimates of ^131^I thyroid doses based on thyroid radioactivity measurements performed within 2 months after the accident and on questionnaire data collected during screening and before diagnosis. Thus, although based on the prevalence data and cross-sectional by design, our study results were not affected by selection or recall bias. All subjects were screened using a standard protocol and screeners were masked to estimates of radiation dose, thus diminishing potential confounding effects of screening and dose-related differential screening. Our findings were further strengthened by the high FNA and surgery compliance rates (95.8% and 91.0%, respectively) and the review of all cases by international pathology experts. Our estimate of EOR was robust and not affected by exclusion of one follicular carcinoma case, cases with simultaneous follicular adenoma or cases with incidental finding of cancer from analysis (EOR/Gy of 2.15, 2.74 and 2.55, respectively).

The study has several limitations. Because only 11 970 of the 36 739 individuals eligible to participate (32.6%) were screened, a possible non-participation bias is a concern. The major reason for non-participation was the difficulty in locating and tracing eligible subjects. Thus, slightly more than 50% of potential subjects could not be traced, primarily because of the high mobility of this young population in the period between the 1986 Chornobyl accident and the start of screening in 1997, and the limited identifying information in the thyroid activity logs through which eligibility was determined. Among those invited into the study, 73.8% participated, which is similar to other large cohort studies (Szklo, 1998) and to the participation rate observed in the parallel study in Ukraine (67.5%) (Tronko *et al*, 2006). About a quarter of those invited to participate did not come for screening, primarily because they lived far from screening centres. Although mobile teams were organised to screen study subjects who lived in areas distant from the stationary screening centres in Minsk or Gomel, this was not possible for areas in other oblasts with sparse distribution of potential study subjects. These areas were also less contaminated after the accident, which could potentially explain the significant differences in dose distribution among participants and non-participants (43% participated among those with thyroid activity measurements 1 Gy and above *vs* 31% with measurements of 0.3–1.0 Gy and 26% of those with measurement <0.3 Gy) (Stezhko *et al*, 2004). However, since screening procedures were uniformly applied to all subjects, any differences in participation were non-differential and would not bias the EOR/Gy estimates for prevalent cases detected during screening. The dose-dependent participation of self-reported pre-screening cases cannot be excluded and could have resulted in higher EOR/Gy estimates. Furthermore, all statistical analyses were adjusted for effects of other potential risk factors such as age, gender and oblast of residence (proxy for iodine deficiency) to eliminate possible effects of differential selection. The annual screening for individuals less than 18 years of age compared to biennial for those more than 18 years may have resulted in increased diagnosis of thyroid cancers among the former. The difference in risk estimates between the two age groups was sizeable although not statistically significant (EOR/Gy of 5.10 for those screened before age 18 years and 1.62 for older study participants, *P*=0.30), but is probably due to higher risks among those exposed at younger ages. Only the central dose estimates were available at the time of analysis and uncertainties in dosimetry have not been taken into account, but their potential impact on the thyroid risk estimates will be evaluated in a later publication.

A recently published parallel study in Ukraine (Tronko *et al*, 2006) observed a higher mean thyroid dose (0.78 *vs* 0.56 Gy). In both studies those younger at the time of exposure tended to have higher risks per unit of dose, whereas the effects of gender were in opposite directions. The estimate of risk among those with doses less than 5 Gy in Ukraine (EOR/Gy=7.35, 95% CI: 2.23–53.11) was much closer in magnitude to the risk estimate for pre-screening cases in our study than to the estimate based on screening-detected cases. This may be due to the heightened awareness in the medical community in Belarus of the potential risk of thyroid cancer among children exposed to the fallout, especially from the heavily contaminated areas in Gomel oblast, and widespread ultrasound screening campaigns in the first 5–10 years after the accident (Ito *et al*, 1997; Astakhova *et al*, 1998). We observed that half of pre-screening cases were among those exposed younger than 5 years old compared with a third among screening cases, and the mean dose of pre-screening cases was significantly higher compared with screening cases (1.77 *vs* 1.08 Gy) but somewhat lower compared to the Ukrainian screening cases (2.00 Gy), supporting the possibility of early detection of cases among those exposed at younger ages from heavily contaminated areas. Only 14 pre-screening cases were reported in the Ukrainian cohort of similar size during the same period of observation and no risk analyses were reported (Tronko *et al*, 2006).

Several studies have investigated risk of Chornobyl-related thyroid cancer among persons exposed as children or adolescents. Case–control studies from the Bryansk oblast of the Russian Federation (Davis *et al*, 2004; Kopecky *et al*, 2006) reported significantly increased risk of thyroid cancer with no variation by gender or age of exposure. A case–control study of thyroid cancer diagnosed in 1987–1992 in children from Belarus (Astakhova *et al*, 1998) found increased risks with dose only among children exposed in Gomel oblast, but in a later study of cases diagnosed in 1992–1998 (Cardis *et al*, 2005) no differences by oblast were observed. Cardis *et al* reported an OR at 1 Gy of 4.9, 95% CI: 2.2–7.5 from a linear-quadratic model over the entire dose range, with evidence of non-linearity at doses above 1.5–2.0. Absence of individual dose estimates and possible underascertainment of thyroid cancer cases from the Cancer Registry of Belarus could be responsible for observed differences in risk estimates with our study (OR at 1 Gy=3.68 from the linear–exponential model).

Our estimates of risk were somewhat lower than those reported from the study of the survivors of atomic bombings in Japan where risk at age 30 was ERR/Gy=5.35 among those exposed younger than 10 and ERR/Gy=4.28 among those exposed between 10 and 19 years old (Preston *et al*, 2007), and much lower compared to the estimate from the pooled analysis of risks after X-ray and *γ*-irradiation (ERR/Gy=7.7, 95% CI: 2.1–28.7) (Ron *et al*, 1995). Our CIs overlap suggesting compatibility of findings. In studies of external irradiation of the thyroid gland, younger age at exposure was significantly associated with increased risk and the evidence for gender was suggestive although not uniform across studies.

Iodine deficiency is thought to increase radioiodine uptake by the thyroid gland and to modify thyroid function after radiation exposure (Robbins *et al*, 2001). Two studies reported an inverse association between iodine deficiency, defined by low urinary iodine excretion levels (Shakhtarin *et al*, 2003) or levels of stable iodine in soil in areas of residence at the time of accident (Cardis *et al*, 2005), and increased risk of radiation-associated thyroid cancer. In the parallel Ukrainian study, place of residence and current iodine excretion did not modify the risk of thyroid cancer (Tronko *et al*, 2006) or the risk of benign follicular adenoma (Zablotska *et al*, 2008). The areas of the northern Ukraine where the study was conducted have more severe iodine insufficiency than the study areas in Belarus, where at the time of screening two-thirds of study participants were iodine deficient with urine levels <100 *μ*g l^−1^ (Table 1). We used four proxy indicators to describe past iodine deficiency and observed that history of diffuse goiter, and diffuse goiter and thyroid volume enlargement diagnosed during screening were significant modifiers of cancer risk. Study participants exposed in Gomel oblast had higher risks compared to subjects from other oblasts (*P*=0.07) and the difference in risk estimates was even more pronounced and significant in the combined analysis of pre-screening and prevalent cases (*P*=0.04). This could be an indication of more severe past iodine deficiency in Gomel oblast. Although the prevalence of goiter at the time of screening in our study was comparable across oblasts, several reports (Kholodova and Fedorova, 1992; Gerasimov, 1993; Gembicki *et al*, 1997) have suggested that iodine deficiency in Gomel oblast around the time of the accident was high and described concerted governmental efforts of iodine supplementation in Gomel 5–10 years after the accident (VanMiddlesworth, 2002).

We did not observe any differences in risk by the size of tumour. To evaluate the effect of referral of smaller nodules sonographically suspicious for malignancy for biopsy, we analysed size of nodule referred for FNA among non-incidental cases (5–10 *vs* ⩾10 mm) and estimated an EOR/Gy of 3.43 (95% CI: not estimated to 464) for the group of smaller nodules (*N*=20 cases) and 1.85 (95% CI: 0.79–4.01) for the larger nodules (*N*=53, *P*>0.50; not shown). The clinical importance of small screening-detected cancers is not well understood and we could not evaluate it in this study based on the first round of screening.

Our findings indicate that in a cohort of children and adolescents from Belarus exposed to the fallout from the Chornobyl accident, thyroid cancer is associated with exposure to ^131^I. Future analyses of incident thyroid cancers identified during additional two screening cycles (2001–2007) should shed light on the clinical importance of screening-detected tumours and age at exposure and time trends effects.

## Figures and Tables

**Table 1:** Descriptive characteristics of prevalent thyroid cases and non-cases

		**Cases**		**Non-cases**				
**Variable**	**Value**	***N***	**%**	***N***	**%**	**Odds ratio^a^**	**95% CI**	***P*-value^b^**
Gender	Men	37	42.53	5602	48.61	1		0.12
	Women	50	57.47	5922	51.40	1.40	0.91–2.15	
								
Age at screening, years	10–14	13	14.90	1379	12.00	1		0.19
	15–17	14	16.10	2161	18.80	0.89	0.41–1.91	
	18–20	12	13.80	2010	17.40	1.03	0.46–2.32	
	21–23	8	9.20	1891	16.40	0.71	0.29–1.75	
	24–26	19	21.80	1840	16.00	1.75	0.83–3.67	
	27–29	15	17.20	1533	13.30	1.73	0.79–3.79	
	30–34	6	6.90	708	6.10	1.61	0.59–4.43	
								
History of nodular goiter	No	55	63.20	11 297	98.00	1		<0.001
	Yes	32	36.80	227	2.00	23.21	14.34–37.57	
								
History of diffuse goiter	No	78	89.70	11 277	97.90	1		<0.001
	Yes	9	10.30	247	2.10	5.15	2.49–10.67	
								
Nodular goiter diagnosed during screening	No	39	44.83	10 895	94.54	1		<0.001
	Yes	48	55.17	629	5.46	19.79	12.65–30.96	
								
Diffuse goiter diagnosed during screening	No	54	62.07	9750	84.61	1		<0.001
	Yes	33	37.93	1774	15.39	3.16	2.02–4.94	
								
Family history of nodular goiter	No	67	77.01	10 649	92.40	1		<0.001
	Yes	20	22.99	875	7.60	3.54	2.13–5.89	
								
Oblast of residence at screening	Minsk	16	18.40	3147	27.30	1		0.54
	Gomel	61	70.10	7110	61.70	1.12	0.63–2.01	
	Other	10	3.50	1267	11.40	1.29	0.58–2.87	
								
Urban status	Urban	39	44.83	7007	60.80	1		
	Rural	48	55.17	4517	39.20	1.44	0.91–2.29	0.12
								
Urinary iodine concentration, *μ*g l^−1^	0–19	10	11.50	1266	11.00	0.79	0.38–1.63	0.47
	20–49	19	21.80	2988	25.90	0.65	0.37–1.17	
	50–99	30	34.50	3236	28.10	1		
	100–299	25	28.70	3426	29.70	0.83	0.49–1.43	
	300+	3	3.40	455	3.90	0.75	0.23–2.48	
	Unknown	0	0.00	153	1.30			

Abbreviation: CI=confidence interval.

a Model adjusted for thyroid dose, age at risk, gender and oblast of residence (Minsk, Gomel, Other).

b *P*-value from the likelihood ratio test evaluating statistical significance of adding corresponding variables to the model.

**Table 2:** Odds ratios and 95% CI by thyroid dose category

**Dose category, Gy**	**Mean dose, Gy**	**Cases (*N*=87)**	**%**	**Non-cases (*N*=11 524)**	**%**	**Odds ratioa,^b^**	**95% CI**
0–0.049	0.02	6	6.9	1985	17.2	1	
0.05–0.14	0.09	11	12.6	2554	22.2	1.42	0.51–3.98
0.15–0.29	0.22	13	14.9	2053	17.8	2.09	0.76–5.75
0.30–0.44	0.37	10	11.5	1281	11.1	2.59	0.90–7.47
0.45–0.64	0.54	10	11.5	1034	9.0	3.27	1.13–9.46
0.65–1.24	0.90	16	18.4	1421	12.3	3.95	1.46–10.69
1.25–2.24	1.64	10	11.5	686	6.0	5.27	1.80–15.46
2.25–4.99	3.18	9	10.3	392	3.4	8.70	2.85–26.55
5.00–32.80	8.84	2	2.3	118	1.0	6.75	1.26–36.22

Abbreviations: CI=confidence interval; Gy=gray.

a Model adjusted for age at risk, gender and oblast of residence (Minsk, Gomel, Other).

b *P*-value of the test of linear trend based on mean values for dose categories=<0.01.

**Table 3:** Risk models and interaction terms for prevalent thyroid cancers

**Modifying factor and its value**	**Cases**	**EOR/Gy^a^**	**95% CI**	***P*-value^b^**
All subjects	85	2.15	0.81–5.47	
				
*Gender*				
Men	36	5.89	1.36–52.72	0.08
Women	49	1.17	0.23–3.70	
				
*Age at the time of the accident, years*				
0–4	26	4.02	0.98–15.07	0.48
5–11	30	1.95	0.41–6.17	
12–18	29	1.40	NE–4.95	
				
*Oblast of residence at the time of the accident*				
Minsk and other	9	2.06	<0–21.89	0.07
Gomel	76	2.51	0.99–5.98	
				
*History of diffuse goiter*				
No	77	1.91	0.67–4.97	<0.01
Yes	8	14.50	4.44–39.03	
				
*Diffuse goiter diagnosed at screening*				
No	52	1.44	0.41–4.04	<0.001
Yes	33	7.83	3.50–17.49	
				
*Urinary iodine concentration,^c^* μ*g l*^−*1*^				
0–19	10	2.40	1.91–7.55	0.30
20–49	19	1.14	NE–4.06	
50–99	29	3.05	NE–7.82	
100+	27	2.17	NE–6.05	
				
*Thyroid volume enlargement* d				
No	71	1.74	0.56–4.68	<0.01
Yes	14	9.10	3.18–22.43	
				
*Max dimension of the tumor after thyroid pathomorphology*				
<10 mm	44	1.35	0.22–5.26	0.30
10+ mm	41	3.40	1.00–13.11	

Abbreviations: CI=confidence interval; EOR/Gy=excess odds ratio per gray; NE=not estimated.

Data restricted to subjects with doses <5 Gy.

a Model adjusted for age at risk, gender and oblast of residence (Minsk, Gomel, Other).

b *P*-value from the likelihood ratio test of homogeneity across categories.

c Excluding 150 subjects with missing urinary iodine concentration.

d Thyroid enlargement was defined as a thyroid volume >18 ml for women and >25 ml for men.

**Table 4:** Models of EOR/Gy and interaction terms for pre-screening and a combined set of pre-screening and prevalent thyroid cancers

	**Pre-screening cases**				**Pre-screening and prevalent cases**			
**Modifying factor and its values**	**Cases**	**EOR/Gy^a^**	**95% CI**	***P*-value^b^**	**Cases**	**EOR/Gy^a^**	**95% CI**	***P*-value^b^**
All subjects	48	8.18	2.11–72.71		133	3.16	1.49–6.95	
								
*Gender*								
Men	26	2.68	NE–74.59	0.88	62	6.27	1.96–30.44	0.13
Women	22	9.09	1.55–103.30		71	2.06	0.75–5.31	
								
*Age at exposure, years*								
0–4	23	4.98	0.91–43.94	0.32	49	2.82	1.04–7.27	0.92
5–7	12	10.44	2.56–76.99		24	3.33	1.14–8.37	
8–18	13	12.24	1.88–109.40		60	3.43	1.23–8.92	
								
*Oblast of residence at the time of the accident*								
Minsk and other	6	10.09	2.70–63.26	0.15	15	1.00	NE–4.19	0.04
Gomel	42	3.41	NE–35.40		118	3.93	1.85–8.65	

Abbreviations: CI=confidence interval; EOR/Gy=excess odds ratio per gray; NE=not estimated.

Data restricted to subjects with doses <5 Gy.

a Model adjusted for age at risk, gender and oblast of residence (Minsk, Gomel, Other).

b *P*-value from the likelihood ratio test of homogeneity across categories.
